# Type of fixation is not associated with range of motion after operative treatment of proximal radius fractures- a systematic review of 519 patients

**DOI:** 10.1016/j.jseint.2024.04.011

**Published:** 2024-04-27

**Authors:** Nadia Azib, Huub H. de Klerk, Remi Verhaegh, Inger N. Sierevelt, Lukas P.E. Verweij, Simone Priester-Vink, Bauke Kooistra, Michel P.J. van den Bekerom

**Affiliations:** a Department of Orthopaedic Surgery, OLVG, Amsterdam, the Netherlands; b Department of Orthopaedic Surgery, Massachusetts General Hospital, Harvard Medical School, Boston, MA, USA; c Amsterdam Shoulder and Elbow Center of Expertise (ASECE), OLVG, Amsterdam, the Netherlands; d Department of Orthopaedic Surgery, University Medical Center Groningen (UMCG) and Groningen University, Groningen, the Netherlands; e Xpert Clinics, Orthopedic Department, Amsterdam, the Netherlands; f Spaarnegasthuis Academy, Orthopedic Department, Hoofddorp, the Netherlands; g Amsterdam UMC, Location AMC, Department of Orthopedic Surgery and Sports Medicine, University of Amsterdam, Amsterdam, the Netherlands; h Amsterdam Movement Sciences, Musculoskeletal Health Program, Amsterdam, the Netherlands; i Medical Library, Department of Research and Epidemiology, OLVG, Amsterdam, the Netherlands; j Department of Orthopedic Surgery, Medische Kliniek Velsen, Velsen-Noord, the Netherlands; k Faculty of Behavioural and Movement Sciences, Vrije Universiteit Amsterdam, the Netherlands

**Keywords:** Proximal radius fractures, Range of motion, Postoperative complications, Revision surgery, Fracture fixation, ORIF, Safe zone definition

## Abstract

**Background:**

The aims of this study are 1) to assess whether open reduction internal fixation (ORIF) techniques for fractures of the proximal radius are associated with the range of motion (ROM), 2) to determine the incidence of hardware-related complications and removal following plate and screw fixation of the proximal radius, and 3) to evaluate whether the safe-zone definition is described in the literature and its relation to the ROM.

**Methods:**

A literature search was performed in the PubMed, Embase, and Cochrane databases. Studies reporting ROM in patients undergoing ORIF for radial head or neck fractures were included. Two treatment groups were defined based on ORIF technique: screws only or plates with and without additional screw placement.

**Results:**

A total of 13 articles were included with 519 patients, of which 271 belonged to the screw group and 248 to the plate group. At final follow-up, the screw group reported a mean supination of 79 (95% CI: 74-83), pronation of 76 (95% CI: 69-84), flexion of 131 (95% CI: 124-138), and loss of extension of 4 (95% CI: 1-7). The plate group reported a mean supination of 72 (95% CI: 65-80), pronation of 697 (95% CI: 60-75), flexion of 126 (95% CI: 118-133), and loss of extension of 7 (95% CI: 1-14).

**Conclusion:**

Predominantly retrospective studies show that the ROM seems similar for screw and plate osteosynthesis of proximal radius fractures. Complication rates are similar as well. The safe-zone definition is rarely reported.

Proximal radial fractures are the most common bony injury to the elbow in adults.^28^ Complex radial head fractures, such as Mason type III and IV, are typically managed operatively, as the radial head functions as a pillar according to the three-column model.^1^^,^^31^ While an isolated radial head fracture is generally considered stable, it is in the event of fragmentation of the radial head or displacement of the radial head fracture fragment, combined with lateral and medial soft tissue injury that instability may play a role.^54^ In the case of instability due to a radial head fracture, it is preferred to reconstruct this lateral column using open reduction and internal fixation (ORIF) or replace it with a radial head prosthesis rather than remove it.^54^

Regarding ORIF, limited research is available on the comparison of different ORIF techniques and hardware type in relation to functional outcomes.^60^ It is theorized that plates, in contrast to countersunk headless screws, are more likely to obstruct the radio-ulnar joint and reduce the pronation-supination arc, especially when the surgeon works outside the so-called ‘safe zone’.^41^ This safe zone is generally described as an area where the radial head does not contact the proximal ulna during maximal pronation and supination.^48^ However, it is specifically this pronation-supination arc that is, required in activities of daily living (ADL). Contemporary tasks, such as using a computer keyboard, opening a door, or using a modern phone, require maximum pronation (65 ± 8 degrees), maximum supination (77 ± 13 degrees), and a maximum flexion-extension arc (142 ± 3 degrees), respectively.^43^ In spite of this, safe-zone definitions are rarely mentioned in studies, and plate removal is commonly seen in such cases due to hardware prominence or misplacement, causing impingement and decreasing rotations.^21^^,^^49^

Therefore, this review aimed to (1) assess whether ORIF techniques (either screws or plating) for fractures of the proximal radius are associated with the final range of motion (ROM), especially forearm rotation, (2) provide an overview of other functional outcomes, complication rates, need for hardware removal, and revision rates following the different ORIF techniques, and (3) evaluate employed definitions of the safe zone and its relationship with ROM across the literature.

## Materials and methods

This study was conducted according to the Preferred Reporting Items for Systematic Reviews and Meta-analyses guidelines.^37^ The protocol is registered at the International Prospective Register of Systematic Reviews under the number: CRD42022380380, and can be accessed electronically at: https://www.crd.york.ac.uk/prospero/.

### Study inclusion and exclusion

Studies reporting ROM in patients undergoing ORIF with (headless) compression screws, neck plates, or head plates for an acute radial head or neck fracture were eligible for inclusion. Studies written in English or Dutch with at least 12 months of mean follow-up and that reported a minimum of 10 patients were included. Reviews, cadaveric studies, expert opinions, abstracts, and surgical technique articles were excluded. Articles in which concomitant fractures were mentioned that could affect elbow ROM significantly were excluded during title-abstract screening. This included ipsilateral, large coronoid fractures, capitellum fractures, olecranon fractures, distal humerus fractures, distal radius fractures, and Galeazzi fractures. A patient with an additional capitellum fracture in the study by Esser et al^14^ was included in the review as the fracture did not influence ROM and the patient had satisfactory results, according to the authors. The small coronoid and capitellum avulsion fractures in the study by Model et al^32^ were also considered noninfluential for ROM, and the article was thus included in the review. Patients undergoing revision surgeries were excluded as well. Studies reporting on both screw and plate fixation had to report outcomes separately for the subgroups to be included.

### Literature search strategy

Relevant studies were identified by searching PubMed, Embase/Ovid, Cochrane Database of Systematic Reviews/Wiley, Cochrane Central Register of Controlled Trials/Wiley, and Web of Science/Clarivate from inception up to October 11th, 2023 (by N.A., R.V., and S.P.V., information specialist). The following terms, including synonyms and closely related words, were used as index terms or free-text words: ‘radius’, ‘fracture’, ‘proximal’, ‘fixation’, and ‘ORIF’. Full search strategies are available as Supplementary Information in Supplementary Data IV. No filters or other restrictions were applied to any of the searches. Title, abstract, and full-text screening were performed by three independent reviewers (N.A., R.V., and H.K.) to identify potentially relevant articles by use of Rayyan.^36^ The authors independently selected articles. Studies were not blinded for author, affiliation, or source. Any disagreements were resolved by a fourth author (M.B.).

### Quality assessment

Two reviewers (N.A. and H.K.) independently assessed each study for its risk of bias using the Cochrane risk-of-bias tool and methodological index for nonrandomized studies (MINORS).^47^ Randomized controlled trials were assessed using the Cochrane risk-of-bias tool (version 2.0; The Cochrane Collaboration, London, UK).^20^^,^^44^^,^^50^ The MINORS is a validated and established index for evaluating the methodological quality of nonrandomized studies. The MINORS involves 12 criteria for comparative studies, of which eight criteria have been designed for noncomparative studies. These items are scored according to the set criteria: 0 (not reported), 1 (reported but inadequate), or 2 (reported and adequate). The MINORS score per group, meaning comparative and noncomparative studies, is displayed as a percentage of the total score. The review of Ekhtiari et al^13^ introduces an interpretation of this score, where a MINORS score ≤ 5 indicated very low-quality evidence, a score of 6-9 indicated low quality of evidence, a score of 10-15 indicated fair quality of evidence, and a score ≥ 16 indicated a relatively good quality of evidence for nonrandomized studies.

### Data extraction

The following parameters were recorded when available: number of patients and elbows, sex, mean age, length of follow-up, Mason fracture type, and ORIF technique. The relevant outcome parameters included primary functional outcomes (including flexion-extension ROM and pronation-supination ROM) and secondary patient-reported outcome measures (PROMs), including visual analog scale (VAS),^9^ Mayo Elbow Performance Score (MEPS),^33^ (quick) Disability of the Arm, Shoulder and Hand Questionnaire,^25^ Oxford Elbow Score (OES),^11^ and Broberg-Morrey score.^6^ The VAS^9^ is a score to measure pain intensity postoperatively, ranging from 0 to 10, with a higher score indicating more pain. The MEPS^33^ is an elbow outcome score used to test limitations in the elbow during ADL. The Broberg and Morrey^6^ rating system describes four sections such as motion, strength, stability, and pain. Both scores range from 0 to 100, with a higher score indicating a superior outcome. The DASH^25^ score uses 30 items to measure physical function and symptoms, whereas the quick DASH uses 11 items to measure the same symptoms. The OES^11^ is a 12-item questionnaire to assess outcomes of elbow surgery based on elbow function, pain, and social-psychological results. Complication and reoperation rates were also collected. The data on the inclusion of heterotopic ossification (HO), post traumatic elbow stiffness, soft tissue injury, and elbow dislocation cases were collected. However, these data were subsequently excluded from analysis due to inconsistent reports.

Two treatment groups were defined based on the ORIF technique. One group comprised patients in whom solely screws were placed, while in the other group a plate was used. Plate variations were not taken into account, as the main goal was to assess whether plates influence the pronation-supination ROM in general terms. Indications for choice of fixation method and ORIF technique were noted when provided.

The complications were categorized as minor and major by two elbow specialists (B.K. and M.B.) based on the severity of the complications. These were in accordance with prior literature.^32^^,^^42^^,^^51^^,^^55^ Major complications were defined as events that seriously affect functioning in a patient’s daily life, are not treatable in a timely manner, and are at least partially irreversible, such as nonunion, secondary displacement, and hardware breakage. These criteria were considered indications for reoperation according to two experienced elbow surgeons (M.B. and B.K.) and definitions aligned with the articles included in this review. All other complications were defined as minor.

For the third aim, a safe zone was identified and extracted within all studies to compare the differences. This was accomplished either by searching for a description of the safe zone based on anatomical landmarks in the section on surgical techniques or by examining the cited sources when outlining the safe zone. These cited sources were explored for safe-zone definitions. If no definition was given in the cited source, the included paper was excluded for this sub analysis.

### Data and statistical analysis

The primary outcome measure was the ROM (including flexion-extension and pronation-supination). Secondary outcomes were PROMs and complication rates.

The data on ROM and PROMs were pooled for the plate and screw groups separately. To enable pooling, data were transformed into means and standard deviations (SD) in case they were reported differently. When medians were reported, transformation to a mean was performed according to Hozo et al^22^ In the case of reported ranges, transformation to SD was done according to Walter et al^52^ and Wan et al.^53^ Chi-squared test was used to compare subgroup differences for Mason types. These results were reported in the following format: X2 (degrees of freedom, N = sample size) = chi-square statistic value, p = *P value.*^61^ Cochran’s Q test was used to compare subgroup differences for complication rates. These results were reported in the following format: (X^2^₁ (degrees of freedom) = Q-test statistic value, p = *P value*).^35^

Pooling of the data was performed based on a random effect model with inverse variance weighting. In this review, the I^2^ statistic was used to explore the level of heterogeneity among the studies. To determine the degree of heterogeneity, thresholds were maintained and categorized as follows: an I^2^ of less than 25% (low heterogeneity), between 25% and 50% (moderate heterogeneity), and over 50% (high heterogeneity) following the guidelines in the Cochrane Handbook and Higgins et al.^18^^,^^19^ Forest plots were created, and I^2^ was calculated using the Meta package in R version 4.2.2 (R Core Team (2022). R: A language and environment for statistical computing. R Foundation for Statistical Computing, Vienna, Austria).^40^

## Results

### Screening and study characteristics

**Figure 1:**
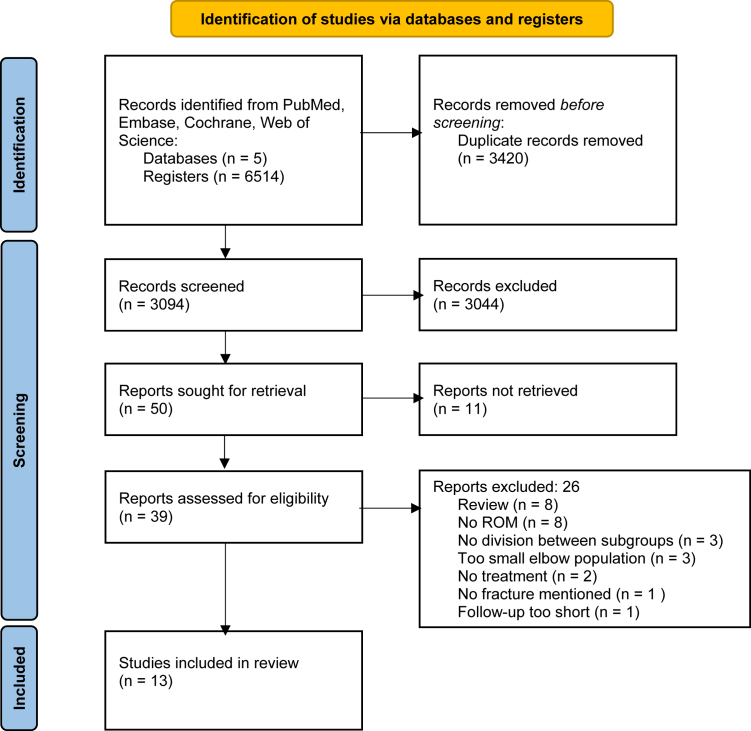
Selection progress flowchart.^37^

**Figure 2:**
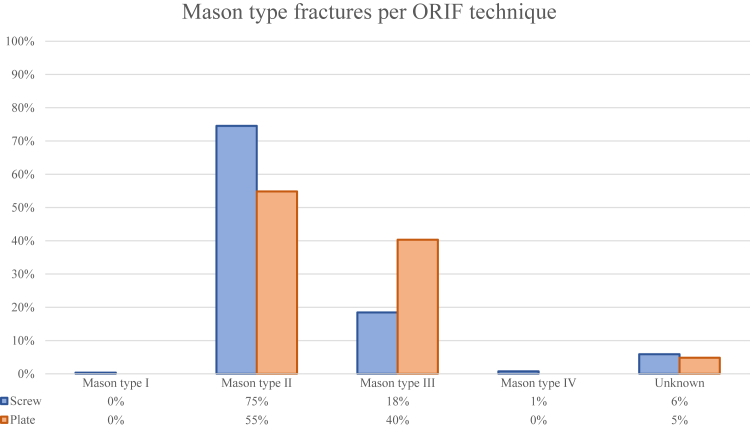
Fracture types categorized based on the Mason classification^23^^,^^31^ and segmented into plate and screw groups, presented as percentages.

The search resulted in 6514 articles, and after duplicates (n = 3420) were removed, 3094 articles were included for title-abstract screening. A total of 50 articles were included for full-text screening. This screening resulted in 13 included articles. The 37 studies excluded during full-text screening were excluded for the following reasons: nonretrievability (n = 11), lack of ROM reports (n = 8), study designed as a review (n = 8), no division between subgroups (n = 3), very small elbow population (n = 3), no treatment (n = 2), no fracture mentioned (n = 1), and very short of a follow-up (n = 1) (Fig. 1). These articles comprised 519 ORIF procedures in a total of 519 patients. The 271 patients in the screw group had a mean follow-up duration of 21 months, while the 248 patients in the plate group had a mean follow-up duration of 22 months.^12^^,^^14^^,^^16^^,^^17^^,^^29^^,^^30^^,^^32^^,^^34^^,^^38^^,^^45^^,^^56^^,^^57^^,^^59^ Within the plate group, higher Mason type fractures were observed in contrast to the screw group (X2 (4, N = 519) = 32.1696, *P* < .001) (Fig. 2). An overview of the baseline study characteristics of the included studies is displayed in Table I.

**Table I:** Study characteristics & outcomes after open reduction internal fixation techniques for fractures of the proximal radius.

ORIF technique	Author, publication year	Study design	No. of subjects	Mason type of fracture	Mean follow-up in months (range)	Mean age in years (range)	Mean postoperative functional outcome	Mean postoperative PROM by study	Minor complications (%)	Major complications (%)	Revisions (%)	Hardware removal (%)	Quality assessment
Screws	Demiroglu et al^12^ 2016	Retrospective	23	II (n = 23)	26 (18-44)	35 (24-53)	S/P ROM 73, 63; F ROM 120; E ROM 8	MEPS/qDASH/VAS 96, 1, 0	7 (20%)	0 (0%)	0 (0%)	0 (0%)	Fair (29/48)
	∗Esser et al^14^ 1995	Retrospective	11	II (n = 11)	48	24 (14-57)	S/P ROM 87, 88; F ROM 142; LoE 1	BM 98	0 (0%)	0 (0%)	0 (0%)	0 (0%)	Fair (24/48)
	Gokaraju et al^16^ 2020	Retrospective	16	Unknown	48	47	S/P ROM 74, 80; F/E Arc ROM 137; ED 6	MEPS/OES/VAS 93, 42, 1	-	-	-	-	Fair (25/48)
	Li et al^29^ 2015	Non-randomized controlled trial	29	II (n = 29)	12	39 (18-56)	S/P ROM 80, 73; F ROM 117; E ROM 10	BM 90	1 (3%)	0 (0%)	0 (0%)	0 (0%)	Fair (28/48)
	Ma et al^30^ 2023 (CCS)	Retrospective	82	II A (n = 34), II B (n = 21), III (n = 27)	12 (11-19)	53 (21-71)	S/P ROM 68, 64; F ROM 127; E ROM -1	MEPS/DASH 89, 12	6 (7%)	7 (9%)	1 (1%)	-	Relatively good (36/48)
	Ma et al^30^ 2023 (HCS)	Retrospective	31	II A (n = 13), II B (n = 13), III (n = 5)	13 (11-21)	52 (26-69)	S/P ROM 68, 64; F ROM 125; E ROM -1	MEPS/DASH 89, 13	3 (10%)	3 (10%)	0 (0%)	-	Relatively good (36/48)
	Model et al^32^ 2022	Retrospective	13	II (n = 6), III (n = 5), IV (n = 2)	72 (21-153)	48 (24-67)	S/P ROM 75, 80; F ROM 139; E ROM -8	qDASH 6	5 (38%)	0 (0%)	0 (0%)	0 (0%)	Low (17/32)
	Mulders et al^34^ 2021	Randomized controlled trial	23	II (n = 23)	12	50 (46-58)	S/P ROM 85, 85; F ROM 140; ED 5	MEPS/DASH/OES/VAS 100, 0, 48, 0	4 (17%)	0 (0%)	0 (0%)	0 (0%)	Some concerns of bias
	Park et al^38^ 2021	Retrospective	9	I (n = 1), II (n = 7), III (n = 1)	21 (16-28)	41 (30-68)	S/P ROM 75,77; F ROM 138; E ROM 1	MEPS 92	-	-	-	-	Relatively good (35/48)
	†Zhou et al^59^ (A), 2022	Retrospective	15	II (n = 11) III (n = 4)	19	30 (16-55)	S/P ROM 86,87 F/E Arc ROM 140	MEPS/qDASH/VAS/ASES 96, 1, 3, 14	0 (0%)	0 (0%)	2 (13%)	2 (13%)	Relatively good (36/48)
	†Zhou et al^59^ (B), 2022	Retrospective	19	II (n = 11) III (n = 8)	20	31 (19-48)	S/P ROM 87, 87 F/E Arc ROM 140	MEPS/qDASH/VAS/ASES 94, 1, 4, 14	0 (0%)	0 (0%)	0 (0%)	0 (0%)	Relatively good (36/48)
Plates	∗Esser et al^14^ 1995	Retrospective	9	III (n = 9)	48	35 (14-57)	S/P ROM 88, 86; F ROM 138; LoE 3	BM 97	1 (11%)	0 (0%)	0 (0%)	0 (0%)	Fair (24/48)
	Gokaraju et al^16^ 2020	Retrospective	12	Unknown	48	47	S/P ROM 68, 74; F/E Arc ROM 130; ED 4	MEPS/OES/VAS 92, 40, 2	-	-	-	-	Fair (25/48)
	Guo et al^17^ 2020	Retrospective	23	III (n = 23)	24	42	S/P ROM 73, 63; F ROM 135; ED 20	MEPS/BM 94, 93	8 (35%)	0 (0%)	0 (0%)	0 (0%)	Fair (31/48)
	Li et al^29^ 2015	Non-randomized controlled trial	29	II (n = 29)	12	39 (18-56)	S/P ROM 72, 62; F ROM 115; E ROM 14	BM 84	3 (10%)	0 (0%)	0 (0%)	0 (0%)	Fair (28/48)
	Ma et al^30^ 2023	Retrospective	57	II A (n = 26), II B (n = 14), III (n = 17)	13 (11-18)	54 (21-73)	S/P ROM 63, 61; F ROM 125; E ROM -1	MEPS/DASH 83, 14	14 (25%)	9 (16%)	2 (4%)	-	Relatively good (36/48)
	Park et al^38^ 2021	Retrospective	2	II (n = 2)	21 (16-28)	41 (30-68)	S/P ROM 80, 65; F ROM 138; E ROM 23	MEPS 98	-	-	-	-	Relatively good (35/48)
	Scoscina et al^45^ 2022	Retrospective	16	III (n = 16)	44	42	S/P ROM 61, 51; F ROM 111; E ROM 30	MEPS/qDASH/BM 84, 29, 75	4 (25%)	8 (50%)	8 (50%)	0 (0%)	Relatively good (33/48)
	Yang et al^56^ (A), 2023	Retrospective	44	II (n = 33) III (n = 11)	19	38	S/P ROM 68, 71; F ROM 125; ED 5	DASH/MEPS/VAS 7, 89, 2	1 (2%)	0 (0%)	0 (0%)	0 (0%)	Relatively good (36/48)
	Yang et al^56^ (B), 2023	Retrospective	46	II (n = 32) III (n = 14)	24	43	S/P ROM 63, 69; F ROM 119; ED 7	DASH/MEPS/VAS 9, 88, 2	8 (17%)	0 (0%)	4 (9%)	4 (9%)	Relatively good (36/48)
	Zarifian et al^57^ 2018	Retrospective	10	III (n = 10)	22	35 (18-60)	S/P ROM 88, 77; F ROM 137; E ROM 178	MEPS/DASH/Oxford score/SF-36 P&M/Grip-strength 94, 8, 45, 57&54, 167 vs. 172	1 (10%)	0 (0%)	0 (0%)	0 (0%)	Relatively good (32/48)

*ORIF*, open reduction internal fixation; *No.*, number; *PROM*, patient-reported outcome measure; *S/*, Supination; *P/*, pronation; *F*, flexion; *E*, extension; *LoE*, loss of extension (reported in degrees); *ED*, extension deficit (reported in degrees); *ROM*, range of motion (reported in degrees); *DASH*, disabilities of arm, shoulder and hand; *MEPS*, Mayo Elbow Performance Score; *OES*, Oxford Elbow score; *BM*, Broberg-Morrey score; *VAS*, Visual Analogue Scale; *SF-36 P&M*, 36-item short form survey physical and mental component; *Grip-strength*, affected elbow vs. spared elbow; *-*, not reported value; *A*, Novel group; *B*, Conventional group; *CCS*, Conventional cortical screw group; *HCS*, headless compression screw group; *ASES*, American Shoulder and Elbow Surgeons rating scale; *qDASH*, Quick Disabilities of the Arm Shoulder and Hand score.

∗ For the study by Esser et al, one patient with Mason type II, fracture had concomitant capitellum fracture, which could influence the ROM.

† Population in study split into a conventional group and a novel group based on different surgical approaches.

### Indication for choice of technique

The choice of ORIF technique was documented when provided by the selected articles. Seven of the 13 (54%) articles^12^^,^^29^^,^^30^^,^^34^^,^^38^^,^^57^^,^^59^ did not provide a justification for their selection of ORIF technique. Of the 6 articles^14^^,^^16^^,^^17^^,^^32^^,^^45^^,^^56^ that described indications, two articles^14^^,^^16^ utilized screws for simple fractures (Mason type II fractures) and plates for fractures with severe comminution or decapitation of the radial head (Mason type III fractures). Model et al^32^ indicated screw fixation for Mason type I fractures with a mechanical block, Mason type II fractures, Mason type III fractures with 3 or less fragments, articular fractures, and extra-articular transverse radial neck fractures. Plate fixation was deemed appropriate in articles reporting on partial fractures,^17^ comminuted fractures with no more than 3 fragments,^17^^,^^45^ young patients,^45^ and small, intra-articular fragments that were difficult to fixate with screws or had a risk of secondary fractures.^56^

### Methodologic quality evaluation

An overview of the methodologic quality evaluations is presented in Supplementary Data I. The Cochrane risk-of-bias tool was used for the randomized controlled trial, resulting in some concerns of bias for Mulders et al.^34^ The MINORS scores for the noncomparative and comparative studies achieved an average of 53% and 65% of the overall score, respectively. Most study designs lacked prospective collection of data (100%), prospective calculation of the study size (100%), and an adequate control group (46%). According to the score by Ekhtiari et al,^13^ 1 article had a low quality of evidence,^32^ 5 articles had a fair quality of evidence,^12^^,^^14^^,^^16^^,^^17^^,^^29^ and 6 articles had a relatively good quality of evidence.^30^^,^^38^^,^^45^^,^^56^^,^^57^^,^^59^

### ROM

**Figure 3:**
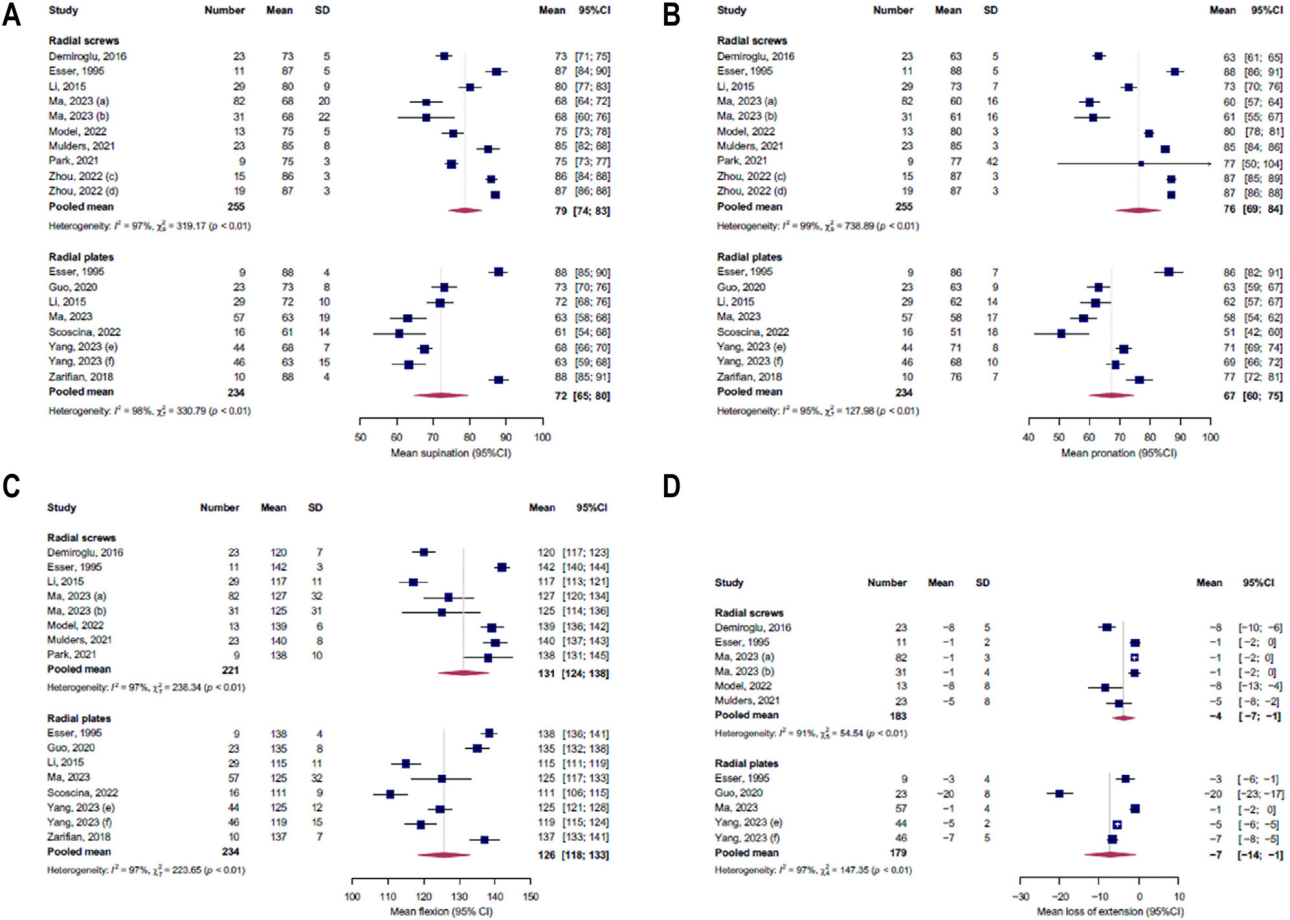
Forest plots demonstrating outcomes per study, divided in groups, for postoperative supination (**A**), pronation (**B**), flexion (**C**), and loss of extension (**D**). Heterogeneity is depicted by I^2^ per subgroup. *Number*, size of the study population, *Mean*, mean range of motion, *SD*, standard deviation, *95%CI,* 95% confidence interval, *I*^2^, level of heterogeneity. *a*, conventional cortical screw group, *b*, headless compression screw group, *c*, novel group, *d*, conventional group, *e*, novel group, *f*, conventional group. The significant variability, potentially influenced by variying study sample sizes and subjectivity of outcome measures, should be taken into account when interpreting the overall findings.

In this study, screw and plate fixation seem similar in outcome regarding ROM. The screw group reported pooled mean values for supination of 79 degrees (95% CI: 74-83), for pronation 76 degrees (95% CI: 69-84), for flexion 131 degrees (95% CI: 124-138), and for loss of extension 4 degrees (95% CI: 1-7). The plate group reported a pooled mean supination of 72 degrees (95% CI: 65-80), pronation of 67 degrees (95% CI: 60-75), flexion of 126 degrees (95% CI: 118-133), and loss of extension of 7 degrees (95% CI: 1-14). The screw group achieved sufficient ROM for ADL regarding supination (at least 77 degrees) and pronation (at least 65 degrees).^43^ The plate group achieved sufficient ROM for ADL regarding pronation and was just short on ROM regarding supination. Both groups lacked ROM for flexion (at least 142 degrees).^43^ An overview of the ROMs per study is presented in Fig. 3.

### PROMs and complications

In this study, seemingly no difference was observed between screw or plate fixation regarding secondary functional outcomes. Complication rates are similar as well. Eleven studies^12^^,^^14^^,^^17^^,^^29^^,^^30^^,^^32^^,^^34^^,^^45^^,^^56^^,^^57^^,^^59^ reported functional outcomes besides the ROM. MEPSs were 94 (95% CI: 91-97) and 89 (95% CI: 83-95) for screw and plate fixation, respectively (Table II). Regarding OES and VAS, no meaningful analysis could be done due to inadequate quality of data and missing values. Forest plots can be found in Supplementary Data II.

**Table II:** Outcomes per group for secondary postoperative outcomes.

Outcome	ORIF technique	No. of studies	No. of patients	Pooled mean *n (95% CI)*	Mean range *(min-max)*	I^2^
MEPS^33^	Screw	5^12^^,^^30^^,^^34^^,^^38^^,^^59^	202	94 (91-97)	89-100	99%
	Plate	4^17^^,^^30^^,^^45^^,^^57^	106	89 (83-95)	83-94	95%
Broberg-Morrey score^6^	Screw	2^14^^,^^29^	40	94 (87-102)	90-96	93%
	Plate	4^14^^,^^17^^,^^29^^,^^45^	77	88 (79-97)	75-97	94%
(q)DASH^25^	Screw	5^12^^,^^30^^,^^32^^,^^34^^,^^59^	206	5 (1-9)	0-13	99%
	Plate	4^30^^,^^45^^,^^56^^,^^57^	173	13 (6-20)	7-27	94%

*ORIF*, open reduction internal fixation; *No.*, number; *95% CI*, 95% confidence interval; *I*^*2*^, Degree of heterogeneity within the specific group; *MEPS*, Mayo Elbow Performance Score; *(Q)DASH*, (Quick) Disabilities of Arm, Shoulder and Hand.

The screw and plate groups were associated with a total of 93 complications, which were reported in 7 studies^12^^,^^14^^,^^29^^,^^30^^,^^32^^,^^34^^,^^59^ for the screw group and 7 studies^14^^,^^17^^,^^29^^,^^30^^,^^45^^,^^56^^,^^57^ for the plate group (Table III). Complication rates were similar between the two groups (X^2^₁ (1) = 1.03, *P* = .31). Of these complications, 66 (71%) were considered minor, with 26 (28%) of those belonging to the screw group and 40 (43%) to the plate group. Regarding major complications, 10 (11%) belonged to the screw group, while 17 (18%) belonged to the plate group. The pooled complication rate for the screw group was 15% (95% CI: 8-27%), and for the plate group 23% (95% CI: 13-38%). Table IV provides details of minor and major complications per group. The most common minor complications were mild pain (n = 18), stiffness (n = 17), and HO (n = 15). The most common major complication was painful arthritis (n = 12).

**Table III:** Outcomes per group for postoperative complications.

Outcome	(Sub)Category	No. of studies	No. of patients	No. of complications	Pooled proportion *(95% CI)*∗	I^2^
Total complication rate	Screw group	7^12^^,^^14^^,^^29^^,^^30^^,^^32^^,^^34^^,^^59^	246	36 Minor: 26 Major: 10	15 (8-27)	45%
	Plate group	7^14^^,^^17^^,^^29^^,^^30^^,^^45^^,^^56^^,^^57^	234	57 Minor: 40 Major: 17	23 (13-38)	79%

*No.*, number; *95% CI*, 95% confidence interval; *I*^*2*^, Degree of heterogeneity within the specific group.

∗ Random effect model.

**Table IV:** Complication rates after open reduction internal fixation techniques for fractures of the proximal radius.

Complications	Occurrence after screws (n = 36) *n (%)*	Occurrence after plates (n = 57) *n (%)*
Minor complications		
Delayed healing	0 (0)	2 (2)
Heterotopic ossification	4 (4)	11 (12)
Infection	1 (1)	1 (1)
Medial epicondylitis	1 (1)	0 (0)
Painless crepitus	4 (4)	0 (0)
Persistent elbow pain	7 (8)	11 (12)
Stiffness	5 (6)	12 (13)
Superficial wound infection	1 (1)	0 (0)
Symptomatic hardware	2 (2)	3 (3)
Transient nerve injury	1 (1)	0 (0)
Total	26 (28)	40 (43)
Major complications		
Hardware breakage	0 (0)	2 (2)
Implant failure	1 (1)	2 (2)
Nonunion	2 (2)	4 (4)
Painful arthritis	7 (8)	5 (6)
Secondary displacement	0 (0)	4 (4)
Total	10 (11)	17 (18)

Percentages displayed are calculated by dividing the number of complications by the total number of complications (n = 93).

Regarding revision surgeries, 2 patients underwent hardware removal in the screw group, and 1 patient underwent revision surgery for nonunion. Fourteen revisions took place in the plate group. Indications for revision surgery in the plate group were hardware removal (n = 4), secondary displacement (n = 4), nonunion (n = 4), and hardware breakage (n = 2). For the studies that reported Mason fracture types, revisions for hardware removal were seen in both Mason type II and Mason type III fractures, while all other revisions were solely attributed to Mason type III fractures.

### Safe-zone definition

**Figure 4:**
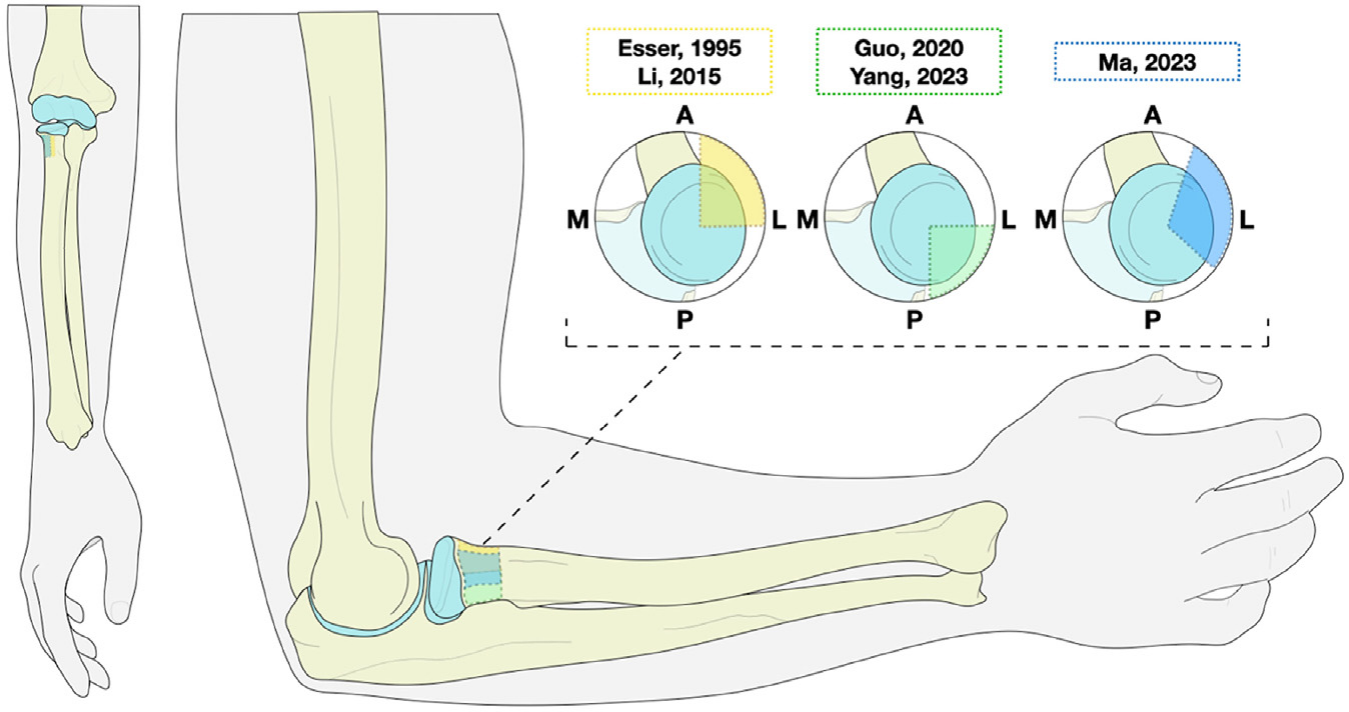
Image depicting described safe zones for the anterolateral aspect (Yellow: Esser et al^14^ & Li et al^29^), posterolateral aspect (Green: Guo et al^17^ & Yang et al^56^) and combined aspect (Blue: Ma et al^30^) of the radial head and neck. *A*, Anteriorl; *L*, Lateral; *P*, Posterior; *M*, Medial.

In this study, plate fixation on the anterolateral aspect of the radial head seems to have a better outcome for supination than plates placed on the posterolateral aspect of the radial head. However, safe-zone definitions currently lack sufficient details in the literature to form a conclusive decision on the optimal placement of plates and their impact on the ROM. Within the screw group, 1 study^29^ described a safe-zone definition. Of the five studies mentioning the usage of a safe zone in the plate group,^14^^,^^17^^,^^29^^,^^45^^,^^56^ four of them provided a clear definition of the safe zone (Fig. 4). Guo et al^17^ and Yang et al^56^ described plate placement on the posterolateral aspect, from Lister’s tubercle to the radial styloid. Plates placed on the posterolateral aspect of the radial head had a combined mean supination of 67 degrees (SD: 8) and a mean pronation of 68 degrees (SD: 3). Li et al^29^ and Esser et al^14^ described placing the screws or plates on the anterolateral aspect of the proximal radius and maximum pronation and supination were confirmed intraoperatively, but did not describe anatomical landmarks for plate placement. Plates placed on the anterolateral aspect of the radial head had a combined mean supination of 76 degrees (SD: 14) and a mean pronation of 68 degrees (SD: 16) (Table V, Fig. 4). Ma et al,^30^ defined the safe zone definition as an arc spanning 41 degrees anteromedially to 69 degrees posterolaterally, with the center positioned at 166 degrees from the great prominence of the bicipital tuberosity. However, they did not provide ROMs for comparison. Instead, MEPS and DASH scores were provided. Plates positioned within the safe zone showed no differences compared to screws. Conversely, outside the safe zone, the average MEPS was notably higher in the screw groups (82 vs. 89 & 90), while the average DASH was lower in the screw groups (15 vs. 9 & 10). A more detailed description of the safe zone can be found in Supplementary Data III.

**Table V:** Supination and pronation range of motions after hardware placement on the anterolateral and posterolateral aspect of the proximal radius.

Safe zone	Study	ORIF technique	ROM supination/pronation *mean (±SD)*∗
Anterolateral	Li et al,^29^ 2015∗	Screws	S/80 degrees (±7) P/73 degrees (±6)
		Plates	S/72 degrees (±1) P/62 degrees (±1)
	Esser et al,^14^ 1995	Screws	S/87 degrees (±5) P/88 degrees (±5)
		Plates	S/88 degrees (±4) P/86 degrees (±7)
Posterolateral	Guo et al,^17^ 2020	Plates	S/73 degrees (±8) P/63 degrees (±9)
	Yang et al,^56^ 2023	Plates (A)	S/68 degrees (±7) P/71 degrees (±8)
		Plates (B)	S/63 degrees (±15) P/69 degrees (±10)

*ORIF*, open reduction internal fixation; *ROM*, range of motion; *SD*, standard deviation, *S/*, supination ROM; *P/*, pronation ROM; *A*, Novel group; *B*, Conventional group.

∗ SD, was estimated when range was given.

## Discussion

### Important findings

Our study aimed to assess if elbow ROM is affected by ORIF technique (either screws or plating) for proximal radius fractures. Additionally, we assessed possible associations with other outcomes and employed safe-zone definitions.

The most important findings were that (1) although patients who underwent plate fixation had higher Mason type fractures, it seems that there are no substantial differences in terms of ROM and secondary functional outcomes compared to the screw group; (2) complication rates were similar between the two groups, while revision surgeries were mostly observed in the plate group (3 vs. 14). Within the plate group, the majority of revision surgeries were associated with Mason type III fractures when Mason type was reported; and (3) the available reports on safe zone definitions lack sufficient details to make conclusive decisions on the optimal placement of plates, as well as their impact on the ROM.

Therefore, our findings suggest that functional outcomes for plate and screw fixation seem similar, regardless of influencing factors such as Mason type. For performing ADLs, both plate and screw fixation demonstrates adequate ROM for pronation. The screw group achieves sufficient supination, while the plate group lacks some degrees in this aspect. Both groups, however, show limitations in ROM with regards to flexion.

### Differences in subgroups

To the best of the authors’ knowledge, other reviews on this subject are not available. Therefore, outcomes found here cannot be compared to outcomes in other literature. According to Cote et al,^8^ a high heterogeneity prevents us from performing statistical subgroup comparisons. This hampers interpretation of overall findings and hinders drawing definitive conclusions regarding the ROMs.

Certain factors that could impact the outcomes, such as complications affecting ROM and the type of plate used, were not taken into account in this review. Notably, plates mentioned in our review were AO miniplates, fracture-specific radial head locking plates, 2.0 mm conventional low-profile mini-T plates (Wego mini low-profile plate system, Wego, Weihai, China), and 2.4 mm locking compression plates (DePuy Synthes. Raynham, MA, USA), low-profile T-plates (DePuy Synthes, Raynham, MA, USA) or undefined plate types. It is important to take these variations into account to understand the impact on the ROM fully. This review did not aim to emphasize subgroup differences but assessed the overall efficacy and safety of plates vs. screws.

Factors like HO, post traumatic elbow stiffness, ligamentous injury, and elbow dislocations were not discussed in this review due to inconsistent reports regarding inclusion and exclusion criteria. HO was not specified in all 13 articles that were included in this review. For post traumatic elbow stiffness, 10 articles did not specify either inclusion or exclusion, and 3 articles excluded patients with a limited ROM preoperatively. Ligamentous injury was included in 3 articles, excluded in 4 articles and unspecified in 6 articles. For elbow dislocations, 10 articles excluded these cases, while 3 articles did not specify either inclusion or exclusion. This hindered the ability to identify subgroups and evaluate the impact of these factors on ROM. Previous literature indicates that the abovementioned factors can limit elbow movement,^4^^,^^15^^,^^46^^,^^58^ and the proper reporting of such factors is therefore important to contextualize the reported ROM. Ideally, a preoperative magnetic resonance imaging should be conducted alongside intraoperative testing to accurately map the extent of all injuries.^10^

Secondly, several authors cite additional factors that could contribute to poorer functional outcomes related to plate fixation. These include pain, injury severity, plate size, and plate placement due to vascularization problems or scar tissue formation postoperatively, and accuracy of reported ROM, which can differ based on the methods used.^2^^,^^5^^,^^29^^,^^38^ Previous literature also suggests that kinesio-phobia and fear of reinjury influences ROM, while clinical and injury-related factors, with the exception of complications, show a weaker correlation with ROM.^24^

Regarding complications, Bauer et al^3^ identified risk factors for HO after ORIF procedure and found that time to first surgery and postoperative mobilization of the elbow are two possible risk factors. Plate fixation might take more time to surgery due to plate positioning, screw placement, plate adaptation and soft tissue handling, causing an increased risk for HO. Our study showed an incidence rate of 15/519 (3%) for HO, while exhibiting a slightly elevated incidence of HO in the plate group (11/248 [4%]), compared to the isolated screw group (4/271 [2%]) (Table III). However, screw fixation might take more time to mobilize due to a lack of stability, especially in the case of comminuted fractures. Both factors could play a big role in avoiding HO and need to be assessed more in future literature.

Lastly, as seen in practice, Mason fracture classification guides treatment choice according to the injury pattern.^23^ Higher Mason types, such as type III and IV, are more likely to be treated by ORIF with plates rather than screws, as demonstrated in our review. This could impact the outcome negatively as the resulting ROM, complications, and revision rates would not be solely decided by the ORIF technique but also by the severity and complexity of the fracture. Literature shows that a more severe Mason type usually correlates with a restricted ROM,^14^ as well as a more ligamentous injury.^26^^,^^27^

Importantly, although numerous factors are described to affect ROM, our review reveals that regardless of these considerations, similar ROM outcomes are achieved for both plate and screw fixation.

### Lack of safe-zone reports

Regarding safe-zone definition, only five of the thirteen studies reported a reproducible definition of the used safe zones for the placement of the hardware. The lack of sufficient details makes it impossible to derive conclusive decisions on the optimal placement of plates, as well as their impact on the ROM. With the limited available data, our findings suggest that plate placement on the anterolateral aspect or the posterolateral aspect of the radial head achieves a similar ROM for pronation. For supination, a slight increase of ROM (9 degrees) was observed when plates were positioned on the anterolateral aspect. However, due to substantial heterogeneity, no statistical analysis could be performed to confirm this. Additionally, a recent study by Ma et al^30^ confirmed that functional scores decrease with plate fixation compared to screw fixation in cases where the fracture cannot be fixated within the safe zone. Conversely, fractures fixated within the safe zone exhibited no differences in outcomes between plate and screw fixation.

The literature describes different methods to identify and use this safe zone for the fixation of radial head and neck fractures. A cadaveric study by Caputo et al^7^ determined the nonarticulating portion of the radial head and neck to ensure safe placement of internal fixation. In full supination, this was considered the posterolateral portion of the radial head, in which a thinner, more yellow cartilage was seen compared to the relatively wide and white cartilage of the articulating portion, indicating thinner hyaline cartilage. Palpation of the radial styloid and Lister’s tubercle identified a safe zone of a 90° angle. Applying these anatomic landmarks to localize a safe zone may assist surgeons in fixating fractures of the radial head and neck without sacrificing ROM.^7^ Hoekzema et al^21^ expanded on this method in their study, concluding that if a proximal radial head plate is placed 166° ± 10° opposite the bicipital tuberosity, the safe zone is still abided by. This landmark can be easily identified on intraoperative imaging.^21^ Smith et al^48^ propose a systematic approach by determining anterior and posterior limits for supination and pronation intraoperatively rather than relying on anatomical landmarks for safe zone identification.^48^ In contrast to plate fixation, hardware prominence is seldom seen in screw fixation, as screws can be buried within the radial head and neck.^39^ In conclusion, describing the utilized method of safe zone identification when fixating fractures with plates is important to facilitate future research on potential differences.

### Limitations

There are also limitations in this review. While this study in part aimed to investigate the impact of plate fixation on ROM, it is crucial to address the presence of selection bias as a notable limitation. As mentioned previously, higher Mason type fractures are usually associated with ligamentous injury and an indication for plate placement. Our study also supported the first notion as plate fixation demonstrated a correlation with higher Mason type. However, our review shows that the mean degrees for ROM seem similar regardless of these factors.

Secondly, studies used within this review were primarily retrospective studies. This, in combination with the inherent bias and significant heterogeneity evident from calculated I^2^ statistics, makes it difficult to draw firm conclusions. The origins of this heterogeneity observed within these studies remain unclear but could be explained by variability in study sample sizes and subjectivity of outcome measures.

Regarding the ROM, reported and measured flexion and extension are vastly different between articles depending on the experience of the observer and the tools used. Conventional clinical goniometry seems to be less accurate in estimating the ROM of the elbow compared to a trained human eye.^5^ Furthermore, extension is reported differently depending on the usage of either “extension” or “loss of extension”, complicating general comparisons.

In reference to the safe-zone definitions, it is important to note that the verification of plate placement was not possible, and descriptions were gathered from the documented methodology. Consequently, caution is warranted in drawing definitive conclusions.

### Future research

Acknowledging the identified limitation regarding selection bias, we propose that future research take the form of prospective studies. Specifically, we advocate for randomized controlled trials comparing screw and plate fixation to further investigate the influence on functional outcomes and the potentially enhanced effect of fracture types and plate fixation on this aspect. Randomized controlled trials also decrease chance of bias and heterogeneity, while comparability across studies can be increased through consistent methodology with respect to data collection, such as ROM measurements and choice of PROMs.

Secondly, it is not always safe to assume that all surgeons use the same safe zone. Therefore, we highly recommend explicitly documenting the safe zone within the methods or surgical techniques section to verify that all individuals follow the same procedure and to discover whether specific outcomes correlate to either technique or patient characteristics. This could be facilitated by implementing a national fracture database, similar to the Dutch database on radial head prostheses.

## Conclusion

This review of predominantly retrospective studies suggests that the ROM seems similar for screw osteosynthesis and plate osteosynthesis of proximal radial fractures, despite the higher Mason types seen in patients treated with plate fixation. Other functional outcomes and complication rates showed similar results as well. Revisions were mostly observed within the plate group; however, the majority of revision surgeries were associated with Mason type III fractures when Mason type was reported. Due to high heterogeneity seen between the studies, statistical analysis was deemed inappropriate.

Finally, safe-zone definitions are rarely reported in literature and often lack sufficient details of operative techniques. This information is essential for gaining perspective on presented outcomes and making conclusive decisions about the optimal placement of plates and their impact on the ROM.

## Disclaimers:

Funding: No funding was disclosed by the authors.

Conflicts of interest: One or more of the authors (H.K.) has received funding from Prof. Michaël-van Vloten Fund Foundation (the Hague, the Netherlands), Van Leersum Grant / KNAW Medical Sciences Fund, Royal Netherlands Academy of Arts & Sciences (Amsterdam, the Netherlands), Anna Fund Foundation | NOREF (Mijdrecht, the Netherlands), The Scholten-Cordes Fund (the Hague, the Netherlands), and the USC Scholarship Foundation (Utrecht, the Netherlands), but this has no relation to this article. N.A. has received funding from “Vreedefonds” and "Hendrik Muller fonds" in The Netherlands, but this has no relation to this article. The other authors, their immediate families, and any research foundations with which they are affiliated have not received any financial payments or other benefits from any commercial entity related to the subject of this article.

## Supplementary data

Supplementary Data II

Supplementary Data III

Supplementary Data IV

## References

[1] Al-Ani Z., Wright A., Ricks M., Watts A.C. (2022). The three-column concept of elbow joint stability and the Wrightington elbow fracture-dislocation classification, emphasizing the role of cross-sectional imaging. Emerg Radiol.

[2] Baghdadi S., Shah A.S., Lawrence J.T.R. (2021). Open reduction of radial neck fractures in children: injury severity predicts the radiographic and clinical outcomes. J Shoulder Elbow Surg.

[3] Bauer A.S., Lawson B.K., Bliss R.L., Dyer G.S.M. (2012). Risk factors for Posttraumatic heterotopic ossification of the elbow: case-control study. J Hand Surg.

[4] Beingessner D.M., Dunning C.E., Gordon K.D., Johnson J.A., King G.J.W. (2005). The effect of radial head fracture size on elbow kinematics and stability. J Orthop Res.

[5] Blonna D., Zarkadas P.C., Fitzsimmons J.S., O’Driscoll S.W. (2012). Accuracy and inter-observer reliability of visual estimation compared to clinical goniometry of the elbow. Knee Surg Sports Traumatol Arthrosc.

[6] Broberg M.A., Morrey B.F. (1986). Results of delayed excision of the radial head after fracture. J Bone Joint Surg Am.

[7] Caputo A.E., Mazzocca A.D., Santoro V.M. (1998). The nonarticulating portion of the radial head: anatomic and clinical correlations for internal fixation. J Hand Surg.

[8] Cote M.P., Lubowitz J.H., Rossi M.J., Brand J.C. (2018). Reviews pooling heterogeneous, low-evidence, high-bias data result in incorrect conclusions: but heterogeneity is an opportunity to explore. Arthrosc J Arthrosc Relat Surg.

[9] Couper M.P., Tourangeau R., Conrad F.G., Singer E. (2006). Evaluating the effectiveness of visual analog scales: a web experiment. Soc Sci Comput Rev.

[10] Cunningham P. (2006). MR imaging of Trauma: elbow and wrist. Semin Musculoskelet Radiol.

[11] Dawson J., Doll H., Boller I., Fitzpatrick R., Little C., Rees J. (2008). The development and validation of a patient-reported questionnaire to assess outcomes of elbow surgery. J Bone Joint Surg Br.

[12] Demiroglu M., Ozturk K., Baydar M., Kumbuloglu O.F., Sencan A., Aykut S. (2016). Results of screw fixation in Mason type II radial head fractures. SpringerPlus.

[13] Ekhtiari S., Horner N.S., De Sa D., Simunovic N., Hirschmann M.T., Ogilvie R. (2017). Arthrofibrosis after ACL reconstruction is best treated in a step-wise approach with early recognition and intervention: a systematic review. Knee Surg Sports Traumatol Arthrosc.

[14] Esser R.D., Davis S., Taavao T. (1995). Fractures of the radial head treated by internal fixation: Late results in 26 cases. J Orthop Trauma.

[15] Frankle M. (1999). Radial head fractures associated with elbow dislocations treated by immediate stabilization and early motion. J Shoulder Elbow Surg.

[16] Gokaraju K., Domos P., Aweid O., Fisher R., White A., Van Rensburg L. (2020). Mid-term outcomes of surgical management of complex, isolated radial head fractures: a multicentre collaboration. Eur J Orthop Surg Traumatol.

[17] Guo L., Li R., Yang X., Yu C., Gui F. (2020). Polylactide pins can effectively fix severely comminuted and unsalvageable radial head fracture: a retrospective study of 40 patients. Injury.

[18] Higgins J., Thomas J., Chandler J., Cumpston M., Li T., Page M. (2022). http://www.training.cochrane.org/handbook.

[19] Higgins J.P.T. (2003). Measuring inconsistency in meta-analyses. BMJ.

[20] Higgins J.P.T., Altman D.G., Gotzsche P.C., Juni P., Moher D., Oxman A.D. (2011). The cochrane collaboration’s tool for assessing risk of bias in randomised trials. BMJ.

[21] Hoekzema N., Gray R., Orbay J., Rubio F., Vernon L., Imada A. (2020). Intraoperative radiographic method of locating the radial head safe zone: the bicipital tuberosity view. J Shoulder Elbow Surg.

[22] Hozo S.P., Djulbegovic B., Hozo I. (2005). Estimating the mean and variance from the median, range, and the size of a sample. BMC Med Res Methodol.

[23] Iannuzzi N.P., Leopold S.S. (2012). In brief: the mason classification of radial head fractures. Clin Orthop.

[24] Jayakumar P., Teunis T., Vranceanu A.-M., Moore M.G., Williams M., Lamb S. (2019). Psychosocial factors affecting variation in patient-reported outcomes after elbow fractures. J Shoulder Elbow Surg.

[25] Jester A., Harth A., Germann G. (2005). Measuring levels of upper-extremity disability in employed adults using the DASH questionnaire. J Hand Surg.

[26] Kaas L., Turkenburg J.L., Van Riet R.P., Vroemen J.P.A.M., Eygendaal D. (2010). Magnetic resonance imaging findings in 46 elbows with a radial head fracture. Acta Orthop.

[27] Kaas L., Van Riet R.P., Turkenburg J.L., Vroemen J.P.A.M., Van Dijk C.N., Eygendaal D. (2011). Magnetic resonance imaging in radial head fractures: most associated injuries are not clinically relevant. J Shoulder Elbow Surg.

[28] Lanzerath F., Hackl M., Wegmann K., Müller L.P., Leschinger T. (2021). The treatment of isolated Mason type II radial head fractures: a systematic review. J Shoulder Elbow Surg.

[29] Li S.L., Lu Y., Wang M.Y. (2015). Is cross-screw fixation superior to plate for radial neck fractures?. Bone Jt J.

[30] Ma S.B., Lee S.K., An Y.S., Choi H.G., Choy W.S. (2023). Is the ‘safe zone’ identified in preoperative computed tomography helpful for choosing optimal implant for fixation of radial head fracture?. Acta Orthop Belg.

[31] Mason M.L. (2005). Some observations on fractures of the head of the radius with a review of one hundred cases. Br J Surg.

[32] Model Z., Merchan N., Rozental T.D., Harper C.M. (2022). Outcomes of radial head fractures treated with the “Tripod technique”. J Hand Surg.

[33] Morrey B.F., An K.N., Chao E.Y.S. (1993).

[34] Mulders M.A.M., Schep N.W.L., De Muinck Keizer R.-J.O., Kodde I.F., Hoogendoorn J.M., Goslings J.C. (2021). Operative vs. nonoperative treatment for Mason type 2 radial head fractures: a randomized controlled trial. J Shoulder Elbow Surg.

[35] (n.d). Cochran’s Q test in SPSS Statistics - procedure, output and interpretation of the output using a relevant example. https://statistics.laerd.com/spss-tutorials/cochrans-q-test-in-spss-statistics.php#:%7E:text=Reporting%20the%20results%20of%20Cochran's%20Q%20test%26text=Cochran's%20Q%20test%20determined%20that,0005.

[36] Ouzzani M., Hammady H., Fedorowicz Z., Elmagarmid A. (2016). Rayyan—a web and mobile app for systematic reviews. Syst Rev.

[37] Page M.J., McKenzie J.E., Bossuyt P.M., Boutron I., Hoffmann T.C., Mulrow C.D. (2021). The PRISMA 2020 statement: an updated guideline for reporting systematic reviews. BMJ.

[38] Park I.-J., Roh Y.-T., Shin S.-H., Park H.-Y., Jeong C., Kang S.-H. (2021). Importance of detection of capitellar cartilage injuries concomitant with isolated radial head fractures: a retrospective clinical study. Acta Orthop Traumatol Turc.

[39] Pearce M.S., Gallannaugh S.C. (1996). Mason type II radial head fractures fixed with Herbert Bone screws. J R Soc Med.

[40] R Core Team (2022). https://www.R-project.org/.

[41] Ries C., Müller M., Wegmann K., Pfau D.B., Müller L.P., Burkhart K.J. (2015). Is an extension of the safe zone possible without jeopardizing the proximal radioulnar joint when performing a radial head plate osteosynthesis?. J Shoulder Elbow Surg.

[42] Sakka S., Coulthard P. (2011). Implant failure: Etiology and complications. Med Oral Patol Oral Cirugía Bucal.

[43] Sardelli M., Tashjian R.Z., MacWilliams B.A. (2011). Functional elbow range of motion for Contemporary tasks. J Bone Jt Surg.

[44] Savović J., Weeks L., Sterne J.A., Turner L., Altman D.G., Moher D. (2014). Evaluation of the Cochrane Collaboration’s tool for assessing the risk of bias in randomized trials: focus groups, online survey, proposed recommendations and their implementation. Syst Rev.

[45] Scoscina D., Facco G., Luciani P., Setaro N., Senesi L., Martiniani M. (2023). Mason type III fractures of the radial head: ORIF, resection or prosthetic replacement?. Musculoskelet Surg.

[46] Shehab D., Elgazzar A.H., Collier B.D. (2002). Heterotopic ossification. J Nucl Med Off Publ Soc Nucl Med.

[47] Slim K., Nini E., Forestier D., Kwiatkowski F., Panis Y., Chipponi J. (2003). Methodological index for non-randomized studies (*MINORS*): development and validation of a new instrument: methodological index for non-randomized studies. ANZ J Surg.

[48] Smith G.R., Hotchkiss R.N. (1996). Radial head and neck fractures: anatomic guidelines for proper placement of internal fixation. J Shoulder Elbow Surg.

[49] Soyer A.D., Nowotarski P.J., Kelso T.B., Mighell M.A. (1998). Optimal position for plate fixation of complex fractures of the proximal radius: a cadaver study. J Orthop Trauma.

[50] Sterne J.A.C., Savović J., Page M.J., Elbers R.G., Blencowe N.S., Boutron I. (2019). RoB 2: a revised tool for assessing risk of bias in randomised trials. BMJ.

[51] Van Delft E.A.K., Vermeulen J., Schep N.W.L., Van Stralen K.J., Van Der Bij G.J. (2020). Prevention of secondary displacement and reoperation of distal metaphyseal forearm fractures in children. J Clin Orthop Trauma.

[52] Walter S.D., Yao X. (2007). Effect sizes can be calculated for studies reporting ranges for outcome variables in systematic reviews. J Clin Epidemiol.

[53] Wan X., Wang W., Liu J., Tong T. (2014). Estimating the sample mean and standard deviation from the sample size, median, range and/or interquartile range. BMC Med Res Methodol.

[54] Watts A.C., Singh J., Elvey M., Hamoodi Z. (2021). Current concepts in elbow fracture dislocation. Shoulder Elbow.

[55] Wu P.H., Shen L., Chee Y.H. (2016). Screw fixation versus arthroplasty versus plate fixation for 3-part radial head fractures. J Orthop Surg.

[56] Yang X., Zhuang J., Xiaosong Z., Huasong W. (2023). Outcomes of radial head fractures treated with pre-curved metacarpal plate. BMC Musculoskelet Disord.

[57] Zarifian A., Rahimi Shoorin H., Hallaj Moghaddam M., Fathi Vavsari M., Gharedaghi M., Moradi A. (2018). The best option in treatment of modified mason type III radial head fractures: open reduction and internal fixation versus radial head excision. Arch Bone Jt Surg.

[58] Zhang D., Nazarian A., Rodriguez E.K. (2020). Post-traumatic elbow stiffness: pathogenesis and current treatments. Shoulder Elbow.

[59] Zhou X., Wang B., Liu Y., Wang Z., Zhao X., Liu F. (2022). Comparative study between the mini-open (≤2.5 Cm) approach and conventional open lateral approach in the surgical treatment of radial head fractures. J Pain Res.

[60] Zwingmann J., Welzel M., Dovi-Akue D., Schmal H., Südkamp N.P., Strohm P.C. (2013). Clinical results after different operative treatment methods of radial head and neck fractures. Injury.

[61] (2018). How to report a chi-square test result.

