# Supplementary material for: Type of fixation is not associated with range of motion after operative treatment of proximal radius fractures- a systematic review of 519 patients
Source: JSES Int. 2024 Apr 27;8(5):1126–36. doi: 10.1016/j.jseint.2024.04.011 (PMC11401575; doi:10.1016/j.jseint.2024.04.011)
Supplement: Supplementary Data II [file mmc1.docx]

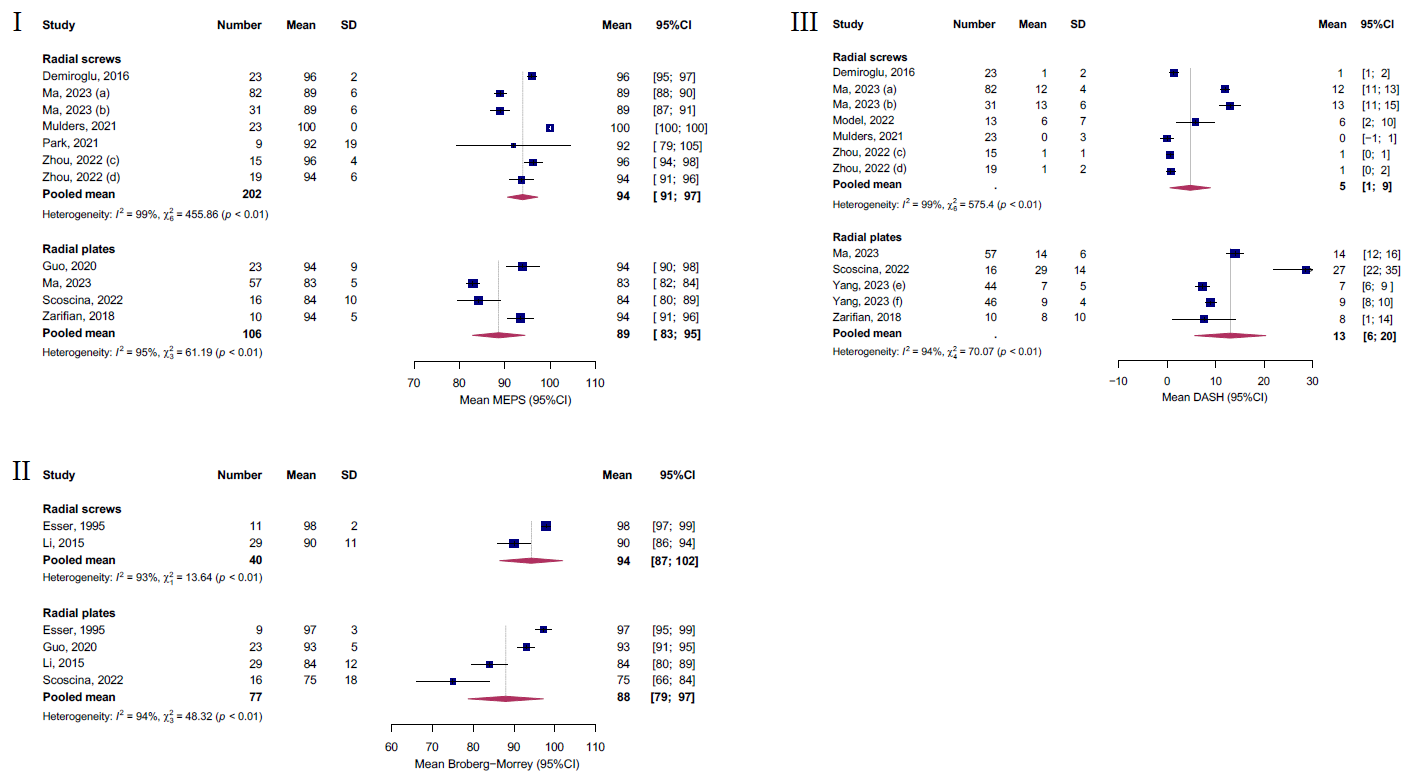
Supplementary Data II: Secondary outcomes per group regarding Broberg-Morrey scores ^6^ (I), DASH ^25^ (II) and MEPS ^33^ (III). Number = size of the study population, Mean = mean range of motion, SD = standard deviation, 95%CI = 95% confidence interval, I^2^ = level of heterogeneity, MEPS = Mayo Elbow Performance Score, (Q)DASH = (Quick) Disabilities of Arm, Shoulder and Hand. a = conventional cortical screw group, b = headless compression screw group, c= novel group, d = conventional group, e= novel group, f = conventional group. The significant variability, potentially influenced by variying study sample sizes and subjectivity of outcome measures, should be taken into account when interpreting the overall findings.
