# Supplementary material for: Type of fixation is not associated with range of motion after operative treatment of proximal radius fractures- a systematic review of 519 patients
Source: JSES Int. 2024 Apr 27;8(5):1126–36. doi: 10.1016/j.jseint.2024.04.011 (PMC11401575; doi:10.1016/j.jseint.2024.04.011)
Supplement: Supplementary Data III [file mmc2.docx]

Supplementary Data III: Reported safe zone definitions.

| **ORIF Technique** | **Author** | **Defined safe zone** |
| --- | --- | --- |
| Screws | Li et al.^29^ | Screws placed in the anterolateral aspect of the radial head to prevent a restricted ROM. Maximum pronation and supination were confirmed intra-operatively. |
|  | Ma et al. ^30^ | The center of the safe zone is 166° from the greatest prominence of the bicipital tuberosity and the arc is approximately 110°. Therefore the radiologic safe zone is defined as the arc involving 41° from anteromedially and 69° from posterolaterally. |
| Plates | Esser et al.^14^ | Plates were placed on the anterolateral aspect of the radial head and neck to prevent restricted supination and pronation. |
|  | Li et al.^29^ | Plates placed in the anterolateral aspect of the radial head to prevent a restricted ROM. Maximum pronation and supination were confirmed intra-operatively. |
|  | Ma et al. ^30^ | The center of the safe zone is 166° from the greatest prominence of the bicipital tuberosity and the arc is approximately 110°. Therefore the radiologic safe zone is defined as the arc involving 41° from anteromedially and 69° from posterolaterally. |
|  | Guo et al.^17^ | Referring to a cadaver study by Caputo et al. for the safe zone, defined as the non-articulating portion of the radial head ^7^. This consists of the posterolateral aspect of the radial head in which the compression screws were buried to prevent limited supination and pronation. The distal radius was used as reference. |
|  | Yang et al.^56^ | Referring to a cadaver study by Smith and Hotchkiss for the safe zone, defined as an arc of 110 degrees of nonarticulating proximal radius, existing at its articulation with the proximal ulna. The zone extends 65 degrees anterior to and 45 degrees posterior from reference marks made with the forearm in neutral rotation ^47^. |
| ORIF = open reduction internal fixation, ROM = range of motion. | | |
