# Supplementary material for: Type of fixation is not associated with range of motion after operative treatment of proximal radius fractures- a systematic review of 519 patients
Source: JSES Int. 2024 Apr 27;8(5):1126–36. doi: 10.1016/j.jseint.2024.04.011 (PMC11401575; doi:10.1016/j.jseint.2024.04.011)
Supplement: Supplementary Data IV [file mmc3.docx]

Supplementary Data IV: Supplemental for article searches.

*All searches were performed on February 29th 2024. No filters were applied.*

**PubMed**

| #1 | ((("Radius"[Mesh] OR radius[tiab] OR radial[tiab]) AND ("Fractures, Bone"[Mesh] OR fractur*[tiab])) OR "Radius Fractures"[Mesh] OR terrible triad[tiab]) | 21,704 |
| --- | --- | --- |
| #2 | (proximal*[tiab] OR head*[tiab] OR neck*[tiab] OR mason[tiab] OR Hotchkiss[tiab] OR (broberg[tiab] AND morrey[tiab])) | 945,878 |
| #3 | (plate*[tiab] OR screw*[tiab] OR fixati*[tiab] OR (("Open Fracture Reduction"[Mesh] OR open reducti*[tiab] OR Open Fracture Reduction*[tiab] ) AND ("Fracture Fixation, Internal"[Mesh] OR internal fixati*[tiab] OR internal fracture fixation*[tiab] OR Fracture Osteosynthes*[tiab] )) OR ORIF[tiab]) | 706,430 |
| #4 | #1 AND #2 AND #3 | 1,981 |

**Embase/OVID**

| 1 | exp radius/ | 16319 |
| --- | --- | --- |
| 2 | radius.ti. or radius.ab. or radial.ti. or radial.ab. | 179301 |
| 3 | 1 or 2 | 182085 |
| 4 | exp fracture/ | 362748 |
| 5 | fractur*.ti. or fractur*.ab. | 375601 |
| 6 | 4 or 5 | 472338 |
| 7 | 3 and 6 | 22577 |
| 8 | exp radius fracture/ | 13361 |
| 9 | terrible triad.ti. or terrible triad.ab. | 290 |
| 10 | 7 or 8 or 9 | 26195 |
| 11 | proximal*.ti. or proximal*.ab. or head*.ti. or head*.ab. or neck*.ti. or neck*.ab. or mason.ti. or mason.ab. or Hotchkiss.ti. or Hotchkiss.ab. or ((broberg.ti. or broberg.ab.) and (morrey.ti. or morrey.ab.)) | 1273320 |
| 12 | plate*.ti. or plate*.ab. or screw*.ti. or screw*.ab. or fixati*.ti. or fixati*.ab. | 893163 |
| 13 | exp open fracture reduction/ | 2615 |
| 14 | open reducti*.ti. or open reducti*.ab. or Open Fracture Reducti*.ti. or open fracture reducti*.ab. | 16704 |
| 15 | 13 or 14 | 17908 |
| 16 | exp osteosynthesis/ | 49694 |
| 17 | internal fixati*.ti. or internal fixati*.ab. or internal fracture fixati*.ti. or internal fracture fixati*.ab. or Fracture Osteosynthes*.ti. or Fracture Osteosynthes*.ab. | 26787 |
| 18 | 16 or 17 | 57838 |
| 19 | 15 and 18 | 12878 |
| 20 | ORIF.ti. or ORIF.ab. | 3500 |
| 21 | 12 or 19 or 20 | 894295 |
| 22 | 10 and 11 and 21 | 2301 |

**Cochrane/Wiley**

*Database of Systematic Reviews & Cochrane Central Register of Controlled Trials*

| #1 | MeSH descriptor: [Radius] explode all trees | 253 |
| --- | --- | --- |
| #2 | radius:ti,ab OR radial:ti,ab | 9110 |
| #3 | #1 OR #2 | 9148 |
| #4 | MeSH descriptor: [Fractures, Bone] explode all trees | 9391 |
| #5 | fractur*:ti,ab | 27070 |
| #6 | #4 OR #5 | 27917 |
| #7 | #3 AND #6 | 2125 |
| #8 | MeSH descriptor: [Radius Fractures] explode all trees | 888 |
| #9 | terrible triad:ti,ab | 9 |
| #10 | #7 OR #8 OR #9 | 2240 |
| #11 | proximal*:ti,ab OR head*:ti,ab OR neck*:ti,ab OR mason:ti,ab OR Hotchkiss:ti,ab OR (broberg:ti,ab AND morrey:ti,ab) | 86794 |
| #12 | plate*:ti,ab OR screw*:ti,ab OR fixati*:ti,ab | 51354 |
| #13 | MeSH descriptor: [Open Fracture Reduction] 1 tree(s) exploded | 72 |
| #14 | open reducti*:ti,ab OR Open Fracture Reducti*:ti,ab | 25716 |
| #15 | #13 OR #14 | 25722 |
| #16 | MeSH descriptor: [Fracture Fixation, Internal] 1 tree(s) exploded | 2233 |
| #17 | internal fixati*:ti,ab OR internal fracture fixati*:ti,ab OR Fracture Osteosynthes*:ti,ab | 3511 |
| #18 | #16 OR #17 | 4303 |
| #19 | #15 AND #18 | 1182 |
| #20 | ORIF:ti,ab | 387 |
| #21 | #12 OR #19 OR #20 | 51414 |
| #22 | #10 AND #11 AND #21 | 92 (1 review, 91 trials) |

**Web of science/Clarative, Core Collection**

TOPIC: (((radius OR radial) AND (fractur*)) OR terrible triad) AND (proximal* OR head* OR neck* OR mason OR Hotchkiss OR (broberg AND morrey)) AND (plate* OR screw* OR fixati* OR ((open reducti* OR Open Fracture Reduction*) AND (internal fixati* OR internal fracture fixation* OR Fracture Osteosynthes*)) OR ORIF)

2140 results

|  | **February 29th 2024** | |
| --- | --- | --- |
| **Database** | **Before deduplication** | **After deduplication** |
| PubMed | 1981 | 1971 |
| Embase | 2301 | 669 |
| Cochrane Database of Systematic Reviews | 1 | 0 |
| Cochrane Central Register of Controlled Trials | 91 | 32 |
| Web of science | 2140 | 748 |
| **Total** | 6514 | 3420 |
