# Supplementary material for: Type of fixation is not associated with range of motion after operative treatment of proximal radius fractures- a systematic review of 519 patients
Source: JSES Int. 2024 Apr 27;8(5):1126–36. doi: 10.1016/j.jseint.2024.04.011 (PMC11401575; doi:10.1016/j.jseint.2024.04.011)
Supplement: Supplementary Data I [file mmc4.docx]

Supplementary Data I: Overview of the methodologic quality evaluations.

| **Comparative Non-randomized (MINORS 1-12)** | **H1** | **N1** | | **H2** | **N**  **2** | | **H**  **3** | | **N**  **3** | **H**  **4** | | **N**  **4** | | **H**  **5** | | **N**  **5** | **H6** | | **N6** | **H7** | | | **N7** | | **H8** | | **N8** | | | **H**  **9** | | **N9** | | **H**  **10** | **N**  **10** | | **H**  **11** | **N**  **11** | **H**  **12** | **N**  **12** | **Total score** |
| --- | --- | --- | --- | --- | --- | --- | --- | --- | --- | --- | --- | --- | --- | --- | --- | --- | --- | --- | --- | --- | --- | --- | --- | --- | --- | --- | --- | --- | --- | --- | --- | --- | --- | --- | --- | --- | --- | --- | --- | --- | --- |
| Park et al. ^38^ | 2 | 2 | | 2 | 2 | | 0 | | 0 | 2 | | 2 | | 0 | | 0 | 2 | | 2 | 2 | | | 2 | | 0 | | 0 | | | 2 | | 2 | | 2 | 2 | | 2 | 1 | 2 | 2 | 35 |
| Demiroglu et al. ^12^ | 2 | 2 | | 2 | 2 | | 0 | | 0 | 2 | | 2 | | 2 | | 2 | 2 | | 2 | 0 | | | 1 | | 0 | | 0 | | | 0 | | 0 | | 2 | 2 | | 2 | 2 | 0 | 0 | 29 |
| Esser et al. ^14^ | 2 | 2 | | 2 | 2 | | 0 | | 0 | 2 | | 2 | | 0 | | 2 | 2 | | 2 | 0 | | | 0 | | 0 | | 0 | | | 0 | | 0 | | 2 | 2 | | 1 | 1 | 0 | 0 | 24 |
| Scoscina et al. ^45^ | 2 | 2 | | 2 | 2 | | 0 | | 0 | 2 | | 2 | | 2 | | 1 | 2 | | 2 | 2 | | | 2 | | 0 | | 0 | | | 0 | | 0 | | 2 | 2 | | 1 | 1 | 2 | 2 | 33 |
| Zarifian et al. ^57^ | 2 | 2 | | 2 | 2 | | 0 | | 0 | 2 | | 2 | | 0 | | 0 | 2 | | 2 | 2 | | | 2 | | 0 | | 0 | | | 0 | | 0 | | 2 | 2 | | 2 | 2 | 2 | 2 | 32 |
| Li et al. ^29^ | 2 | 2 | | 2 | 2 | | 0 | | 0 | 0 | | 2 | | 2 | | 2 | 2 | | 2 | 0 | | | 0 | | 0 | | 0 | | | 0 | | 0 | | 2 | 2 | | 2 | 2 | 0 | 2 | 28 |
| Guo et al. ^17^ | 1 | 1 | | 2 | 2 | | 0 | | 0 | 2 | | 2 | | 0 | | 0 | 2 | | 1 | 2 | | | 2 | | 0 | | 0 | | | 2 | | 2 | | 2 | 2 | | 1 | 1 | 2 | 2 | 31 |
| Gokaraju et al. ^16^ | 2 | 2 | | 2 | 2 | | 0 | | 0 | 2 | | 2 | | 2 | | 1 | 2 | | 2 | 0 | | | 0 | | 0 | | 0 | | | 0 | | 0 | | 2 | 2 | | 1 | 1 | 0 | 0 | 25 |
| Zhou et al. ^59^ | 2 | 2 | | 2 | 2 | | 0 | | 0 | 2 | | 2 | | 0 | | 0 | 2 | | 2 | 2 | | | 2 | | 0 | | 0 | | | 2 | | 2 | | 2 | 2 | | 2 | 2 | 2 | 2 | 36 |
| Yang et al. ^56^ | 2 | 2 | | 2 | 2 | | 0 | | 0 | 2 | | 2 | | 0 | | 0 | 2 | | 2 | 2 | | | 2 | | 0 | | 0 | | | 2 | | 2 | | 2 | 2 | | 2 | 2 | 2 | 2 | 36 |
| Ma et al. ^30^ | 2 | 2 | | 2 | 2 | | 0 | | 0 | 2 | | 2 | | 0 | | 0 | 2 | | 2 | 2 | | | 2 | | 0 | | 0 | | | 2 | | 2 | | 2 | 2 | | 2 | 2 | 2 | 2 | 36 |
| **Non-comparative Non-randomized (MINORS 1-8)** | **H1** | **N1** | | **H2** | **N**  **2** | | **H**  **3** | | **N**  **3** | **H**  **4** | | **N**  **4** | | **H**  **5** | **N**  **5** | | | | | | **H6** | **N6** | | **H7** | | **N7** | | | **H8** | | **N8** | | **Total score** | | |  |  |  |  |  |  |
| Model et al. ^32^ | 2 | 2 | | 2 | 2 | | 0 | | 0 | 2 | | 2 | | 0 | 1 | | | | | | 2 | 2 | | 0 | | 0 | | | 0 | | 0 | | 17 | | |  |  |  |  |  |  |
| **Cochrane risk of bias tool for RCT's** | **H**  **1** | | **N**  **1** | | | **H**  **2** | | **N**  **2** | | | **H**  **3** | | **N**  **3** | | | **H**  **4** | | **N**  **4** | | | **H**  **5** | | | | **N**  **5** | | |  |  |  |  |  |  |  |  |  |  |  |  |  |  |
| Mulders et al. ^34^ | Low | | Low | | | Low | | Some concerns | | | Low | | Low | | | Low | | Low | | | Low | | | | Low | | |  |  |  |  |  |  |  |  |  |  |  |  |  |  |
